# Muslim Travelers’ Inconvenient Tourism Experience and Self-Rated Mental Health at a Non-Islamic Country: Exploring Gender and Age Differences

**DOI:** 10.3390/ijerph18020758

**Published:** 2021-01-18

**Authors:** Heesup Han, Soyeun Lee, Antonio Ariza-Montes, Amr Al-Ansi, Beenish Tariq, Alejandro Vega-Muñoz, Su-hyun Park

**Affiliations:** 1 College of Hospitality and Tourism Management, Sejong University, 98 Gunja-Dong, Gwanjin-Gu, Seoul 143-747, Korea; heesup@sejong.ac.kr (H.H.); lsy2you82@hotmail.com (S.L.); amralansi1@gmail.com (A.A.-A.); 2 Social Matters Research Group, Universidad Loyola Andalucía, C/Escritor Castilla Aguayo, 4, 14004 Córdoba, Spain; ariza@uloyola.es; 3 Department of Marketing, Institute of Business Administration (IBA), Karachi 75270, Pakistan; binish_sh@live.com; 4 Faculty of Business and Administration, Universidad Autónoma de Chile, Santiago 7500912, Chile; 5 Department of Hotel & Leisure Management, Pai Chai University, 155-40 Baejae-ro, Seo-gu, Daejeon 35345, Korea; kayla9252@gmail.com

**Keywords:** muslim travelers, inconvenient tourism experience, self-rated mental health, value from hedonic experience, gender, age, SEM

## Abstract

This research examined international Muslim travelers’ intention formation of a non-Islamic country. Our proposed theoretical framework encompassing inconvenient tourism experience, mental health, hedonic value experience, and satisfaction included a sufficient level of predictive power for intent. These variables played a vital role in increasing intention, whereas an inconvenient tourism experience decreases self-rated mental health and hedonic value. Our result also provided meaningful information that boosting Muslim travelers’ mental health, hedonic experience, and satisfaction is essential for minimizing the effect of the inconvenient tourism experience. In addition, gender and age have been shown to play a moderating role in affecting behavioral intention.

## 1. Introduction

Muslim tourism has considerably grown in the global tourism market over the past decades [1,2,3,4]. In 2019, Muslim tourism has contributed USD 180 billion to the global economy. It is further expected to grow to USD 300 billion in 2020 [5]. In the highly competitive international tourism marketplace, the fast-growing Muslim tourism is nowadays considered as an important niche market [6,7,8]. Many non-Islamic countries are paying attention to this emerging Muslim tourism sector, making diverse endeavors to boost the increase of the number of overseas Muslim travelers [3,7]. For many countries, supporting Muslim tourism can be the way to stay competitive and increase market share in the global tourism industry [2,6]. Hence, numerous destinations are becoming aware of the importance of Muslim tourism and are becoming active in offering diverse benefits/advantages and pleasurable experiences to international Muslim visitors, which are not likely to be available in other rival destination countries [7,9].

Nonetheless, Muslim travelers’ inconvenient tourism experiences at a non-Islamic country are quite common [1] due to the lack of understanding of Muslim culture, Islamic hospitality notions, and the principals of Islam [10]. Olya and Al-Ansi [11] asserted that service guidelines, facilities (e.g., prayer room), halal foods, and halal-friendly products are limited/barely available for Muslim visitors in the hotels, restaurants, and tourist sites of many non-Islamic destinations. This deficiency of Islamic tourism attributes possibly lowers mental health, pleasurable experience, and fulfilment among Muslims traveling to a non-Islamic country [6,7,9]. According to recent researches, international Muslim tourists show a strong preference to the destination where halal-friendly tourism attributes/environment are likely available and easily accessible, which allow them to engage in tourism behaviors that meet their religious norms [1,9,11,12].

Undoubtedly, the global tourism market sees Muslim tourism as a lucrative sector [3,10]. To take full advantage of Muslim tourism in a non-Islamic country, a clear understanding of the needs and behavior of Muslim travelers is required. Yet, little research has examined Muslim tourists’ tourism needs and behaviors while traveling to a non-Islamic country. Bearing this in mind, the current study is developed in the Republic of Korea. In addition, to motivate Muslim tourists to travel abroad, minimizing the possible inconveniences that they experience while traveling is one of the essential requites [1]. However, scant research has investigated the effect of the inconvenient tourism experiences on Muslim travelers’ mental health, hedonic value, satisfaction, and the development of behavioral intentions for non-Islamic destinations. Moreover, male and female travelers often have different levels of interest on tourism products/attributes at an international tourism destination [13,14]. Females are more interested in beautification products (e.g., skin care, plastic surgery, cosmetics, and clothing) than males, whose traveling needs are more related to relaxing, business, or health check [13,15]. Although the preferences and tourism behaviors of males and females are dissimilar, fewer studies have been conducted on gender differences in overseas Muslim travelers’ decision formation and behaviors. Further, international travelers’ behaviors often differ based on their age [16]. Indeed, previous research revealed that the age group difference exists in shaping the tourism behaviors [17,18]. However, scant research has yet to examine the age difference on international Muslim travelers’ behaviors.

To fill this void, this study aimed to investigate the causal associations among Muslim travelers’ perceived inconvenience of tourism experience, self-rated mental health, value from hedonic experience, and satisfaction, as well as to examine whether gender and age play a moderating role in the potential influence of such relationships on behavioral intentions towards non-Islamic destinations. In addition, this study sought to discover the mediating role of mental health, hedonic value, and satisfaction, for Muslim traveler’s tourism experience and behavioral intention. The subsequent sections include the current literature review, study methods, and results. Finally, Section 5 presents the discussion and implications.

## 2. Literature Review

### 2.1. Inconvenience of Tourism Experience Among Muslim Travelers

Although not considerably studied in the current tourism literature, one’s inconvenient experiences when traveling to a certain destination is crucial in explicating his/her decision formation and tourism behaviors [6,19]. Individuals often perceive the inconvenience of their tourism experience at the international destination [1,3,11]. Such perception of inconvenience is considered a cognitive dimension in the traveler/customer behavior [20,21]. According to Han et al. [19], in the tourism sector, individuals’ inconvenience perception refers to tourists’ cognitive assessment of the severity of the possible difficulties that may arise while traveling to specific destinations or consuming goods/services at those destinations. Overseas tourists normally have a variety of tourism experiences when engaging in tourism activities at the international destination; and in turn, such tourists also experience some difficulties during their trip, which decline their pleasurable/comfortable travel experience and induce anxiety [6]. Han et al. [19] asserted that such difficulties are the major facet of overseas tourists’ inconvenient tourism experiences.

The recent literature emphasizes the criticality of tourists’ inconvenience perception and its considerable influence on their tourism behaviors [1,19]. The inconvenience of the tourism experience that international Muslim tourists perceive can be far more serious than that of other traveler types [6]. Specifically, at non-Islamic destinations where the Muslim-friendly tourism environment and infrastructure are not sufficiently built, such inconvenience is maximized [10]. Undeniably, the tourism environment and attributes of many non-Islamic destinations are not well equipped for the international Muslim travelers [3,11]. Accordingly, it is likely that Muslim travelers’ perceived the inconvenience of tourism experience at many tourist sites of non-Islamic destinations as severe, affecting their overall tourism experience and behaviors in an unfavorable way for the destinations [1,11].

Diverse cognitive factors (e.g., inconvenience while traveling, tourism attributes performances, overall quality, tourism environments, tourism product value) determine tourists’ mental health and their effect (e.g., hedonic value experience) [9,14,22,23,24]. Recent studies revealed that travelers’ perceived inconvenience is a cognitive variable link to their mental health/well-being and hedonic experience in their intention generation process for a tourism product [9,12,19]. Al-Ansi et al. [1] indicated that Muslim tourists’ difficulties are often derived from halal foods, prayer facilities, social environment, locals’/other travelers’ attitude, and lack of cultural understanding. Minimizing the difficulties is an excellent way to increase convenient tourism experiences among Muslim travelers, which eventually enhances their mental health and hedonic value experience [3,6]. Taking this into account in the current study, it is plausible that the perceived inconvenience of Muslim travelers’ tourism experience when traveling to non-Islamic destinations negatively influences the self-rated mental health and hedonic value experience.

**Hypothesis 1** **(H1).** *Perceived inconvenience of tourism experience has a negative impact on self-rated mental health among international Muslim travelers.*

**Hypothesis 2** **(H2).** *Perceived inconvenience of tourism experience has a negative impact on value from hedonic experience among international Muslim travelers.*

### 2.2. Self-Rated Mental Health

Mental health is undoubtedly a rising problem in every business segment with a rapidly increasing number of humans suffering from mental health related problems [25,26]. The mental health problems that individuals are struggling with include stress, anxiety, concern, emotional depression/disorder, and self-distrust [27]. Han et al. [28] defined a self-rated mental health as “individuals’ self-assessment of their own present condition/status of mental health”. If a person’s mental health is excellent, they tend to have low levels of mental stress/depression/anxiety and have a high self-trust and confidence [25]. In the field of tourism, Han et al. [28] asserted that travelers often get benefits from the quality travel experience in terms of reduced fatigue/stress and boosted well-being. They found that such increased mental health helps the travelers’ overall tourism experience to be pleasurable/enjoyable and satisfactory. Their assertions are consistent with Hwang and Lee’s [29] arguments that tourists’ mental health often leads to improved cognitive and affective tourism experiences. These researchers agree that the mental health of travelers has become an increasingly important subject in the contemporary international tourism industry. Based on this, it is posited that Muslim travelers’ self-rated mental health includes hedonic value experience and satisfaction.

**Hypothesis 3** **(H3).** *Self-rated mental health has a positive impact on value from hedonic experience among international Muslim travelers.*

**Hypothesis 4** **(H4).** *Self-rated mental health has a positive impact on satisfaction among international Muslim travelers.*

### 2.3. Value from Hedonic Experience

Due to its importance, academics in tourism and consumer behavior actively seek a comprehensive understanding of the value that customers’ experience [30,31]. Patrons’ purchase decisions/motivations are highly linked to the value that they can obtain from consumption activities [31,32]. Hedonic value experience refers to the possible acquiring of the emotional or affective advantages/benefits during the consumption procedure of a particular product/service such as pleasure, joy, and excitement [30,33]. Individuals perceive the hedonic facet of the product/service value when having enjoyment/entertainment experiences [32]. This hedonic value experience is often considered to be subjective and private [34]. The scope of the hedonic value experience comprises consumers’ needs for enjoyable and interesting experiences when consuming the product/service, which are mainly related to the affective nature [31,34].

The existing tourism literature and consumer behavior literature have consistently dealt with the relationship between pleasure value experience, satisfaction, and intention. [31,32,35]. Several studies have shown that consumers evaluate the hedonic phase of the product/service/brand experience in their satisfaction evaluation and post-purchase decision-making processes [32,35]. Consumers that prefer the affective/emotional aspect of the product/service/brand experience often weigh on the hedonic value in their satisfaction and intention generation [31,33,35]. Based on the prior studies, we posit that Muslim tourists’ hedonic value experience positively influences their satisfaction and behavioral intentions for a destination.

**Hypothesis 5** **(H5).** *Value from hedonic experience has a positive impact on satisfaction among international Muslim travelers.*

**Hypothesis 6** **(H6).** *Value from hedonic experience has a positive impact on behavioral intentions among international Muslim travelers.*

### 2.4. Traveler Satisfaction

In the tourism and consumer behavior, satisfaction is one of the most influential factors on customer behavior [21,36]. Oliver [21] regarded customer satisfaction as the criterion for evaluating customer product/service consumption experiences [21]. Consistently, in the context of international tourism, traveler satisfaction indicates the assessment of a traveler’s overall tourism experience in a particular overseas destination [37,38]. Traveler satisfaction is particularly crucial in inventing efficient service/marketing tactics for a tourism product or fortifying the existing tactics [36,38,39]. Therefore, this concept often interests industry practitioners and academics in various tourism sectors [39,40]. The existing tourism literature deals extensively with the impact of traveler satisfaction [36,37,39]. For instance, Lai [39] empirically demonstrated the considerable effect of satisfaction on traveler behavioral intentions. In addition, Byun and Jang [38] found that traveler satisfaction triggers positive post-purchase decisions for a brand/destination. These studies provided evidence that travelers’ satisfactory experience with the consumption situation of a tourism product eventually enhances the behavioral intentions, leading to affirmative post purchase actions for the product/destination. Given this, it would be true that Muslim travelers’ satisfaction with their tourism experience at a destination determines the level of behavioral intentions for the selection of a tourism destination in the future.

**Hypothesis 7** **(H7).** *Traveler satisfaction exerts a positive influence on the intentions of action of international Muslim travelers.*

### 2.5. Behavioral Intentions

A traveler’s strong intentions for a specific action are believed to likely result in the actual behavior [38,41,42]. Hence, behavioral intentions are regarded as the fundamental concept in traveler behavior and marketing [21]. Numerous researchers in the social psychology sector claimed that individuals’ various behaviors are predictable by identifying their intentions for the behaviors [38,41,42,43]. Oliver [21] described such behavioral intentions as one’s sturdy likelihood and readiness of performing specific behaviors. The behavioral intentions can be either favorable or unfavorable [43] and often include such dimensions as the intention to revisit and recommend [14,20,44]. In the present research, behavioral intentions refer to Muslim travelers’ likelihood/readiness to travel to a non-Islamic destination repeatedly and to provide word-of-mouth about their tourism experience at the destination. A high repurchase and word-of-mouth in the tourism sector frequently reflects the traveler’s positive behaviors for a firm, and are essential requisites for the firm’s survival and financial success [14].

### 2.6. Gender and Its Influence

One of the most important personal characteristics can be gender [16,45]. Therefore, many researchers have examined the gender difference in diverse contexts such as consumer behavior, tourism, marketing, psychology, and education. Previous studies have shown that gender difference is evident in consumers’ decision-making process and behaviors [46,47,48,49,50]. In consumer behavior, Meyers-Levy and Maheswaran [49] indicated that women tend to extensively engage in the processing of information, yet male customers do not proactively engage in such information processing. Compared to female consumers, men are less likely to concentrate on the consumption process, and their decision is less likely to be influenced by their emotion/affect/mood [13,46,51]. Mittal and Kamakura [52] identified that when the satisfaction level is similar, the repeat patronage of female clienteles is greater than that of male clienteles. Homburg and Giering [47] claimed that male customers’ decision to purchase is mainly triggered by their satisfaction experiences with the product and its functionality, whereas female customers’ purchase decision is heavily triggered by the sale process. In the hospitality sector, Oh et al. [50] asserted that at the similar level of product performances, female guests in a hotel tend to build a higher expectation than that of male guests. In the recent hospitality sector, Hwang et al. [14] demonstrated that customers’ perception, mental well-being, attitudes, satisfaction, and intention are significantly influenced by gender. Consistently, Hwang and Kim [16] uncovered that gender significantly influences the intent generation process of hospitality patrons. Therefore, in line with the existing literature, this study proposes: Given this evidence, it is conceivable that Muslim travelers’ post-purchase decision for a tourism destination and its predictors are affected by gender difference.

**Hypothesis 8a–g** **(H8a–g).** *Gender plays a significant moderating role in forming behavioral intentions among international Muslim travelers.*

### 2.7. Age and Its Influence

Another critical factor of personal characteristics can be age [13,16,48]. This demographic factor along with gender is believed to be the most frequently employed personal characteristics factor as its impact on buying behavior is not trivial but consequential [13,17,18,48]. Researchers frequently group customers’ age ranges into high and low (or old and young) categories [16]. Studies of the effects of age have often focused on the dissimilarity of cognitive and emotional assessments of a product/service and the presentation of its qualities. In consumer behavior, Yoon [53] indicated that young customers often seek diverse information sources in making a purchasing decision, whereas senior customers frequently prefer the heuristic process when making a purchase-related decision. Homburg and Giering [47] also indicated that while the available information provides the base for young individuals’ purchase decision and behavior, the existing experiences with a product/service normally become the base for old consumers’ decision-making process and consumption behavior. Although people have a similar degree of cognition and emotion, older and younger customers undergo different selection processes and show different purchasing behaviors [13]. The dissimilar needs of product/service attributes’ performances and the difference in decision/loyalty/attachment formation and behaviors across age categories have been observed in many existing studies in diverse contexts [17,54]. Based on the aforementioned pieces of evidence, it is plausible that Muslim tourists’ intention generation process is influenced by age difference.

**Hypothesis 9a–g** **(H9a–g).** *Age plays a significant moderating role in forming behavioral intentions among international Muslim travelers.*

### 2.8. Proposed Theoretical Framework

**Figure 1:**
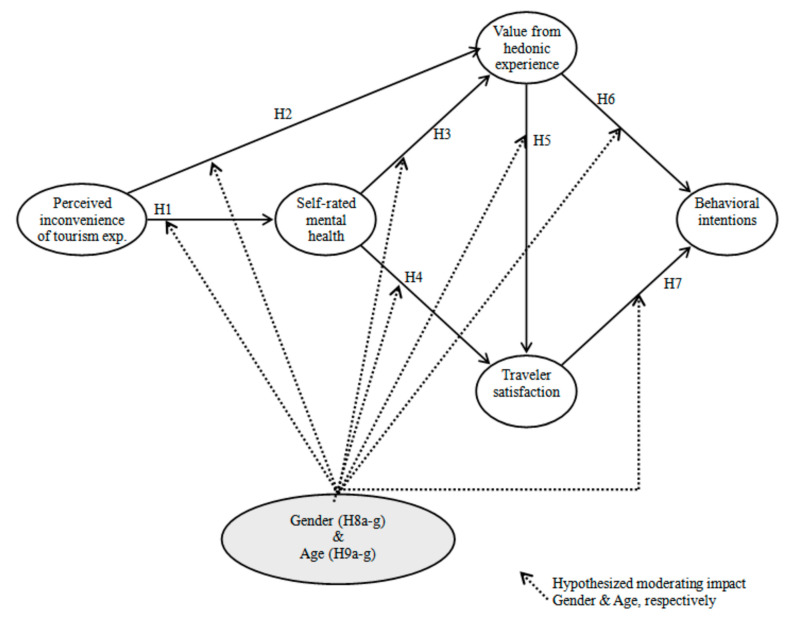
The proposed theoretical framework.

Figure 1 displays a suggested conceptual framework. The theoretical framework includes four major drivers of action intentions by international Muslim travelers to non-Islamic destinations (i.e., perceived inconvenience of tourism experience, self-rated mental health, the value from the hedonic experience, and traveler’s satisfaction). This model contains seven research hypotheses related to these variables (Hypotheses 1–7). In addition, Hypotheses 8a–g and 9a–g were incorporated into the proposed model to examine the moderating effects of gender and age.

## 3. Methods

Structural equation modeling (SEM) seems an appropriate choice to analyze the model, which consists of multiple direct, indirect, and moderating relationships [55]. Since our study has multiple relationships to be analyzed, this research utilized SEM AMOS for the effective obtainment of our research objectives.

### 3.1. Identification of Inconvenient Tourism Experience Factors

A series of qualitative person-to-person interviews were conducted to explore the discomfort Muslim travelers may experience when traveling to a non-Islamic country. The interviewees were actual international Muslim visitors traveling to non-Islamic countries. They have various travel purposes such as pleasure, medical treatment (or healthcare), education, cultural experience, and business. Through this interview process and the associated literature search [1,6,12], we uncovered a total of five major inconvenience items, namely halal-friendly, halal facilities, halal foods, service/information, halal-friendly attire of staff/tourists/locals, and halal-friendly attitude of staff/tourists/locals. These given items were utilized to measure the perceived inconvenience of tourism experience among international Muslim tourists at a non-Islamic destination.

### 3.2. Measures for Self-Rated Mental Health, Value, Satisfaction, and Intention

The validated measures for self-rated mental health, value from hedonic experience, traveler satisfaction, and behavioral intentions are adopted in the existing studies [21,25,28,43]. We utilized multiple items and a 7-point scale. In particular, three items were used to assess self-rated mental health. We also used three items to evaluate the value from hedonic experience. For the assessment of traveler satisfaction, two items were used. In addition, behavioral intentions were measured in three items. These measurement items along with the measure for the inconvenience of tourism experience were included in the survey questionnaire (see Appendix A).

### 3.3. Data Collection Process

An early version of the survey contained an explanation of this study, the measures for research variables, and demographics of the participants. This survey questionnaire was improved based on pretesting and feedback from tourism scholars. This survey questionnaire was improved based on pretesting and feedback from tourism scholars.

The survey questionnaire has been improved on the basis of a pre-test and feedback targeted at tourism scholars.

It was then further refined and confirmed based on reviews from academic and industry experts. A visitor’s survey was performed. Muslim visitors traveling to Korea were the study population. A non-probability convenience sampling approach was used. In particular, we conducted a survey on popular tourist destinations (e.g., Itawon, N Seoul Tower, Gyeongbokgung Palace, Myeong-dong) in the Republic of Korea where there are many Muslim travelers and other international travelers often visit and engage in diverse tourism activities. The survey was conducted in the middle of May 2019 for about 2 weeks. The developed questionnaire was disseminated to Muslim travelers. Specifically, the well-trained graduate students who are from Islamic countries or have a good understanding of Islamic culture approached Muslim travelers, and requested them for survey participation. The surveyor briefly explained the research description and survey purpose to those international Muslim travelers who had agreed on survey participation. The questionnaire was filled out and returned onsite. The surveyors had obtained 290 valid returns, which are analyzed for the study results.

### 3.4. Sample Characteristics

Among the 290 survey participants, a total of 139 respondents (47.9%) were male, whereas 151 respondents (52.1%) were female. The average age of the participants was 33.77 years. Most of the participants reported visiting Korea for the first time (77.6%), followed by 2–3 times (15.5%), six times or more (3.8%), and 4–5 times (3.1%). In addition, most of the respondents reported that their travel purpose was for pleasure (68.6%). Regarding the participants’ income level, about 67.3% indicated under USD 40,000 (67.3%), followed by between USD 40,000–69,999 (21.9%), between USD 70,000–99,999 (6.8%), and USD 100,000 or more (4.0%). The survey participants are in general highly educated. In addition, 46.2% of the participants are pursuing or have completed graduate level studies, while 29.2% of them are pursuing or have completed a master’s degree. About 58.7% reported that they are single, whereas 40.9% indicated that they are married.

## 4. Results

### 4.1. Confirmatory Factor Analysis

Prior to conducting a structural analysis, we tested the model’s fitness by the confirmatory factor analysis using SPSS Amos 20 (IBM, New York, NY, US). The statistics showed a good model fit (χ^2^ = 262.671, *df* = 92, *p* < 0.001, χ^2^/*df* = 2.855, Root Mean Square Error of Approximation (RMSEA) = 0.080, Comparative Fit Index (CFI) = 0.953, Incremental Fit Index (IFI) = 0.954, Tucker-Lewis Index (TLI) = 0.939). Table 1 includes the measurement model evaluation results in detail. The composite reliability (CR) values (perceived inconvenience of tourism experience = 0.868, self-rated mental health = 0.925, value from hedonic experience = 0.948, traveler satisfaction = 0.937, behavioral intention = 0.865) were all above the minimum threshold of 0.70 [55], which confirms the internal consistency in scale items. Then, the convergent validity was measured by examining the average variance extracted (AVE) value. Our result showed that all the values (perceived inconvenience of tourism experience = 0.572, self-rated mental health = 0.805, value from hedonic experience = 0.859, traveler satisfaction = 0.882, behavioral intention = 0.684) were above 0.500 [55], confirming good convergent validity. Moreover, the AVE values are higher than the corresponding inter-measure correlations (squared) (see Table 1), which demonstrate possession of sufficient discriminant validity [55].

### 4.2. Structural Model Evaluation and Hypotheses Testing

As mentioned earlier, structural equation modeling (SEM) was used to test and analyze the proposed hypotheses. Overall, the fit statistics showed a good model fit (χ^2^ = 275.526, *df* = 96, *p* < 0.001, χ^2^/*df* = 2.870, RMSEA = 0.080, CFI = 0.951, IFI = 0.951, TLI = 0.939). In addition, this proposed theoretical framework showed a satisfactory level of anticipation for behavioral intentions. It accounted for 54.1% of the total variance in behavioral intentions. Subsequently, the hypothesized effect of perceived inconvenience of the tourism experience on self-rated mental health and value from hedonic experience was assessed. Our results indicated that the perceived inconvenience of tourism experience had a significant impact on self-rated mental health (β = −0.291, *p* < 0.01), which supported Hypothesis 1. Yet, its relationship with the value from hedonic experience was insignificant (β = −0.036, *p* > 0.05), therefore Hypothesis 2 was not supported.

The results of Hypothesis 3 were supported, showing that self-rated mental health had a positive and significant impact on the value of the hedonistic experience (β = 0.643, *p* < 0.01). The findings have shown that self-rated mental health has a positive and significant impact on the value of hedonic experience (β = 0.643, *p* < 0.01). On the other hand, the effect of self-assessed mental health on satisfaction was not significant. Therefore, Hypothesis 4 was not supported (β = 0.060, *p* > 0.05).

**Figure 2:**
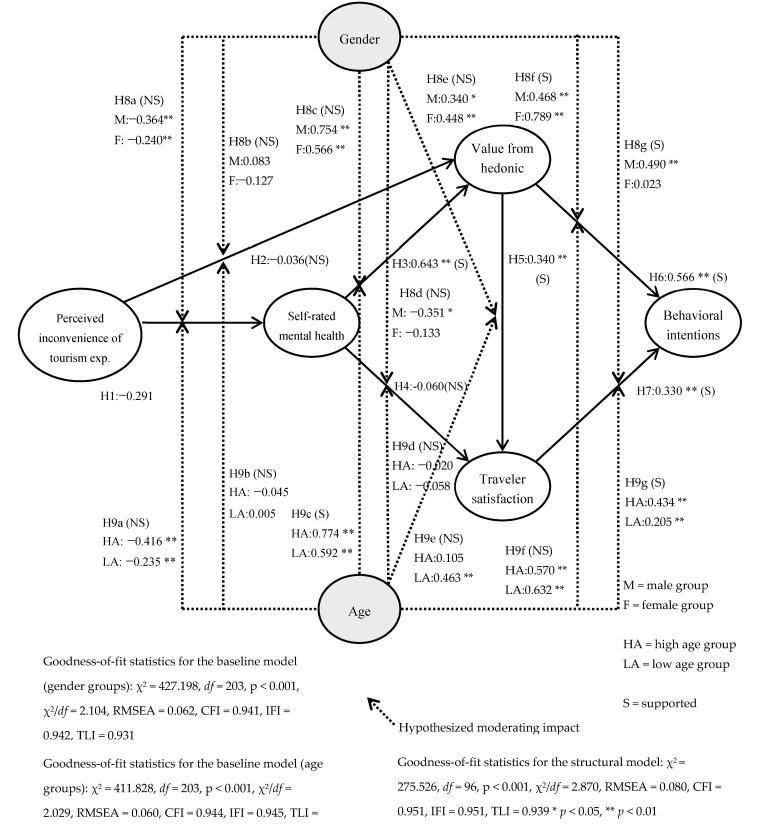
The structural model evaluation and baseline model estimation.

The proposed influence of value from the hedonic experience was assessed. As expected, the value from the hedonic experience significantly affected traveler satisfaction (β = 0.340, *p* < 0.01) and behavioral intentions (β = 0.566, *p* < 0.01), supporting Hypotheses 5 and 6. Finally, the linkage between traveler satisfaction and behavioral intentions was significant (β = 0.330, *p* < 0.01), supporting Hypothesis 7. Figure 2 and Table 2 show the structural model evaluation results in detail.

An indirect influence research construct was then investigated. The result of the indirect effect assessment showed that the value from hedonic experience, self-rated mental health, and perceived inconvenience of tourism experience (β = −0.146, *p* < 0.01) significantly exerted an indirect impact on Muslim travelers’ behavioral intention for non-Islamic destinations. That is, traveler satisfaction, value from hedonic experience, and self-rated mental health acted as important mediators in the proposed model. A total effect of the research variables was examined. It was found that the value from hedonic experience had the utmost impact on behavioral intentions, followed by self-rated mental health, traveler satisfaction, and perceived inconvenience of tourism experiences. This result proves that the hedonistic value experience is considered relatively important among Muslim travelers when deciding on behavioral intentions for a non-Islamic destination.

### 4.3. Baseline Model Evaluation and Gender Difference Testing

To investigate the possible moderating effect of gender, a multi-group analysis was performed to compare the difference in coefficients of the corresponding paths for males and females using a Chi-square test. First, all the responses were separated by gender, with 139 travelers each in the male group and 151 travelers in the female group. The fit statistics showed a good model fit (χ^2^ = 427.198, *df* = 203, *p* < 0.001, χ^2^/*df* = 2.104, RMSEA = 0.062, CFI = 0.941, IFI = 0.942, TLI = 0.931). Table 3 and Figure 2 show the results in detail. We compared the baseline model for gender groups to a set of models where specific pathways of interest were constrained to be equal. This process was to uncover how differently male and female Muslim travelers’ inconvenient tourism experience affects its outcome variables.

The invariance test disclosed that the associations between hedonistic values and behavioral intentions, as well as between traveler satisfaction and behavioral intentions were significantly dissimilar across two gender groups. Therefore, the results support Hypotheses 8f and 8g. Yet, the other associations were not different significantly with gender difference, which did not support Hypotheses 8a–8e.

### 4.4. Baseline Model Evaluation and Age Difference Testing

The baseline models for age groups were generated. All the responses were split into high and low age groups. There are 113 travelers in the old age group and 177 travelers in the low age group. The fit statistics showed a good model fit (χ^2^ = 411.828, *df* = 203, *p* < 0.001, χ^2^/*df* = 2.029, RMSEA = 0.060, CFI = 0.944, IFI = 0.945, TLI = 0.934). Table 4 and Figure 2 present the results in detail. The base line model for the age groups was compared to the nest structural models with the constraint of one particular path by employing the Chi-square test. This process was to unearth how the associations among the research variables differ based on age groups.

The invariance test disclosed that the paths from self-rated mental health to hedonic value and from traveler satisfaction to behavioral intentions differed significantly by age group. Hence, Hypotheses 9c and 9g were supported. Yet, the paths from perceived inconvenience to self-rated mental health, from perceived inconvenience to hedonic value, from self-rated mental health to satisfaction, from hedonic value to satisfaction, and from hedonic value to behavioral intentions did not differ significantly. Therefore, the results did not support Hypotheses 9a, 9b, and 9d–9f.

## 5. Discussion and Implications

There is very limited academic research evaluating the causal relationship between variables that induce Muslim tourists to form post-purchase intentions for non-Islamic destinations, taking into account the moderating impact of Muslim tourist demographic characteristics. The current research has filled this existing gap. This research successfully explored the apparent role of inconvenient tourism experience, self-rated mental health, value from hedonic experience, and satisfaction in generating behavioral intentions. Our hypothesized framework, including these variables, fully explained the total variance of intentions. Additionally, to better understand the Muslim traveler’s decision-making process for non-Islamic destinations, moderating gender and age turns out to be fundamental. Other demographic variables, such as level of education/income or marital status, were also taken into account for analysis, but no more significant interventional effects were found. This result helps destination practitioners create efficient strategies for increasing travelers’ retention and word-of-mouth activities, advancing the extant knowledge regarding what triggers Muslim travelers’ positive responses/behaviors for the destination. The non-Islamic destination tourist sites hardly contain guidelines (e.g., hotels, halal restaurants, prayer areas, shopping places, and hospitals) deemed important for Muslim tourists to decide on tourism destinations. It may happen due to our lack of understanding about the unique needs of Muslim tourists. For example, a limited number of such goods/service and area/facilities were accessible to Muslims traveling to the destination. Overall, the current study offered a better comprehension of Muslim tourism in the international tourism industry.

Consistent with the findings of prior studies [1,19], Muslim travelers’ perceived that the inconvenience of tourism experience contained a vital role in the behavioral intention generation process, directly influencing self-rated mental health and value from hedonic experience. Therefore, it is apparent that the decision of whether Muslim travelers will revisit their destination and speak positively is largely dependent on the degree and influence of the inconvenience they perceive while traveling to a non-Islamic destination. In order to minimize Muslim travelers’ inconvenience experiences that decrease their mental health and behavioral intentions, destination practitioners should direct their tactics toward a halal-friendly tourism environment. Specifically, destination practitioners should make every endeavor to offer quality halal foods by increasing its availability, providing halal-friendly services, making halal facilities and halal information available, and improving staff/locals’ halal-friendly attitude. This effort can contribute to effectively inducing favorable post-purchase decisions and better promoting a non-Islamic destination product to international Muslim travelers, which eventually brings the increased market share of halal tourism.

Based on our findings, the total effect of value from hedonic experience on behavioral intentions was much greater than that of other study structures. Understanding the criticality of hedonic value experience in the intention generation process for a non-Islamic destination, destination practitioners need to focus on boosting Muslim travelers’ hedonic value through offering pleasant, enjoyable, and interesting tourism experiences at the destination. Jeaheng et al. [6] asserted that boosting the performance of tourism products, encounter services, and physical environment are essential for increasing travelers’ hedonic experience. Therefore, enhancing the tourism product performance, service-encounter performance, and physical environment performance at tourist sites/places can be excellent methods that help Muslim travelers feel pleasant, enjoyable, and interested while traveling. As evidenced in this research, such efforts for performance enhancement will ultimately contribute to obtaining a high satisfaction and behavioral intention among Muslim travelers.

Our findings demonstrated that gender moderates the associations between hedonic value and behavioral intentions, as well as between traveler satisfaction and behavioral intentions. The female group was stronger in the association between hedonistic values and intentions, and the male group was stronger in the association between satisfaction and intention. This is in line with previous studies [14,47,49,50,52] and findings that gender plays a significant moderating role in the intent generation process of consumer behavior.

The result implies that when experiencing a similar level of hedonic value, female travelers generate stronger behavioral intentions than that of male travelers, and that when experiencing the same level of satisfaction, male travelers make solider intentions than female travelers. From the theoretical perspective, given that scant research has examined the possible gender difference among overseas Muslim tourists, the present research successfully broadened our comprehension about the role of gender. From a practical perspective, a development of useful strategies is necessary by considering that this gender difference, as the dissimilarity of the value, satisfaction, and intention associations across genders, is evident. To efficiently drive female Muslim travelers’ behavioral intentions to a non-Islamic country, destination practitioners should center on providing diverse hedonic experiences to them (pleasant, enjoyable, and interesting tourism experiences). Meanwhile, to elicit male Muslim travelers’ intentions, practitioners need to focus on boosting their satisfaction level while traveling.

The finding of this research revealed that in the relationships between self-rated mental health and hedonic value and between satisfaction and behavioral intentions, there were significant moderating effects with age. Such results are similar to those of Chua et al. [13] and Chang and Chen [54], who demonstrated the moderating role of age in tourists’ intention generation process. The high age group was stronger than the low age group in the relationship between self-rated mental health and hedonic value (high age group: β = 0.774, *p* < 0.01 vs. low age group: β = 0.592, *p* < 0.01), and also in the relationship between the satisfaction and intention (high age group: β = 0.434, *p* < 0.01 vs. low age group: β = 0.205, *p* < 0.01). These results imply that, given a similar level of mental health and satisfaction, older travelers have stronger hedonistic value experiences and build stronger behavioral intentions than lower aged travelers. This result of the age difference includes critical theoretical and practical meaning. Theoretically, given that the existing research paid much attention to the age dissimilarity and its role in the Muslim traveler decision-making process. The present research successfully broadened our comprehension regarding the moderating role of age. Specifically, this study considerably adds knowledge to the international Muslim tourism literature by deepening the mental health—hedonic value relationship and the satisfaction—intention association with the age influence. Practically, as these relationships have a dissimilar level of strength across age groups, the different tactics of strengthening the relations for each age group should be invented and used. For instance, to successfully enhance hedonic value experience and behavioral intentions among high age group travelers, dealing with their self-assessed mental health and satisfaction may be important.

Self-rated mental health was uncovered as the vital trigger of hedonic value experience and satisfaction in forming intentions, in which the results are inconsistent with the previous literature in the field of tourism [28,29]. Being aware of the importance of self- assessed mental health, destination practitioners should make diverse endeavors on enhancing Muslim travelers’ mental health perception through reducing their feeling of anxiety/stress/depression and increasing their feeling of calm/comfort. Some recent studies claimed that green items/places and atmospherics offer fundamental resources for individuals’ mental health and induce their well-being perception [27]. Accordingly, placing green items (e.g., flowers, plants), increasing green spaces for rest/leisure, and designing green atmospherics in tourist sites can be effective to help international Muslim travelers feel mentally healthy, comfortable, and relaxed while traveling. As demonstrated in this research, this effort for mental health increase will result in the improved value perception and traveler satisfaction.

The crucial mediating nature of traveler satisfaction, hedonic value experience, and self-rated mental health were uncovered. Within our theoretical framework, each mediator increased the effect of the preceding factor(s) on the outcome variable(s). That is, when these mediators are involved, the impact of the explanatory variables(s) on the explained variable(s) is taken full advantage of. This finding offered destination researchers and practitioners critical information that involving these variables with a mediating nature into the conceptual framework are essential to better understand the role of research variables in international travelers’ behavioral intention formation. Tourism academics need to comprehend the mediating characteristics of traveler satisfaction, hedonic value experience, and self-rated mental health when building a theoretical model and extending the existing traveler behavior models using these mediators. Our results further informed destination practitioners that dealing with Muslim traveler satisfaction, hedonic value experience, and mental health is a useful tactic to take full advantage of minimizing their inconvenient tourism experiences in inducing favorable behavioral intentions toward the destination. The research by Cohen and Avieli [56] addressed the role of food that could be attractive or disruptive in the tourism experience. Experiencing authentic cuisine is certainly a great gastronomic delight for every tourist, but unfamiliar local food and inadequate culinary establishment can also cause tourists to experience anxiety, irritation, and frustration [56]. These two-faced foods are more important to Muslim tourists [12] as they can only eat halal food or food prepared halal-friendly by the rules of Islam [1,12]. Knowing and taking into account the needs of Muslim tourists such as halal food, public awareness of their cultures, attires, and facilities (prayer rooms) can have a positive impact on their hedonic experience and mental health, which will affect the satisfactory Muslim tourism experience.

Although this study made efforts to enrich the current understanding of Muslim tourism, a number of limitations should be noted. First, the current research contained a generalization problem. That is, the survey of this study was conducted with foreign Muslim travelers in South Korea, which is a non-Islamic destination. Therefore, generalizing the findings should be cautious. Future research should include samples from more non-Islamic countries. Second, representativeness of Muslim tourists are insufficient as the sample (*N* = 290) is not very large. Third, other important factors exist, for example, destination image, destination involvement/attachment, destination familiarity, affecting travelers’ post-purchase behaviors for a destination, which have not been incorporated into the proposed theoretical framework. The explanatory power of the proposed model can be enhanced when involving such constructs. For future research, an empirical endeavor should be made to expand the hypothesized framework for a better prediction of behavioral intentions among international Muslim travelers.

## Figures and Tables

**Table 1:** The measurement model evaluation.

	(1)	(2)	(3)	(4)	(5)	Mean	Standard Deviation (SD)	Construct Reliability (CR)	Average Variance Extracted (AVE)
(1) Perceived inconvenience of tourism experience	1.000	-	-	-	-	3.669	1.269	0.868	0.572
(2) Self-rated mental health	−0.297 ^a^ (0.088) ^b^	1.000	-	-	-	4.995	1.230	0.925	0.805
(3) Value from hedonic experience	−0.237 (0.056)	0.614 (0.049)	1.000	-	-	5.409	1.181	0.948	0.859
(4) Traveler satisfaction	0.079 (0.006)	0.139 (0.019)	0.275 (0.076)	1.000	-	4.207	1.479	0.937	0.882
(5) Behavioral intentions	−0.086 (0.007)	0.433 (0.187)	0.630 (0.397)	0.456 (0.208)	1.000	5.220	1.224	0.865	0.684

Note. Goodness-of-fit statistics for the measurement model: χ^2^ = 262.671, *df* = 92, *p* < 0.001, χ^2^/*df* = 2.855, RMSEA = 0.080, CFI = 0.953, IFI = 0.954, TLI = 0.939. ^a^ Correlations between constructs. ^b^ Squared correlations.

**Table 2:** The structural model evaluation.

Hypotheses	Links			β	*t*-Values
Hypothesis 1	Perceived inconvenience of tourism experience	→	Self-rated mental health	−0.291	−4.486 **
Hypothesis 2	Perceived inconvenience of tourism experience	→	Value from hedonic experience	−0.036	−0.673
Hypothesis 3	Self-rated mental health	→	Value from hedonic experience	0.643	11.161 **
Hypothesis 4	Self-rated mental health	→	Traveler satisfaction	−0.060	−0.699
Hypothesis 5	Value from hedonic experience	→	Traveler satisfaction	0.340	3.772 **
Hypothesis 6	Value from hedonic experience	→	Behavioral intentions	0.566	9.515 **
Hypothesis 7	Traveler satisfaction	→	Behavioral intentions	0.330	5.743 **
Indirect effect on behavioral intentions:		Total effect on behavioral intentions:		Variance explained:	
β value from hedonic experience = 0.112 * β self-rated mental health = 0.417 ** β perceived inconvenience of tourism experience = −0.146 **		β traveler satisfaction = 0.330 ** β value from hedonic experience = 0.678 ** β self-rated mental health = 0.417 ** β perceived inconvenience of tourism experience = −0.146 **		R^2^ for behavioral intentions = 0.541 R^2^ for traveler satisfaction = 0.093 R^2^ for value from hedonic experience = 0.429 R^2^ for self-rated mental health = 0.085	

Note. Goodness-of-fit statistics for the structural model: χ^2^ = 275.526, *df* = 96, *p* < 0.001, χ^2^/*df* = 2.870, RMSEA = 0.080, CFI = 0.951, IFI = 0.951, TLI = 0.939. * *p* < 0.05, ** *p* < 0.01.

**Table 3:** The invariance model assessment for gender groups.

Paths	Male Group (*n* = 139)		Female Group (*n* = 151)		Baseline Model (Freely Estimated)	Nested Model (Equally Constrained)
	β	*t*-Values	β	*t*-Values		
Perceived inconvenience of tourism experience → Self-rated mental health	−0.364	−3.984 **	−0.240	−2.702 **	χ^2^ (203) = 427.198	χ^2^ (204) = 427.665 ^a^
Perceived inconvenience of tourism experience → Value from hedonic experience	0.083	1.086	−0.127	0.091	χ^2^ (203) = 427.198	χ^2^ (204) = 430.958 ^b^
Self-rated mental health → Value from hedonic experience	0.754	9.207 **	0.566	7.582 **	χ^2^ (203) = 427.198	χ^2^ (204) = 430.011 ^c^
Self-rated mental health → Traveler satisfaction	−0.351	−2.449 *	0.133	1.405	χ^2^ (203) = 427.198	χ^2^ (204) = 430.700 ^d^
Value from hedonic experience → Traveler satisfaction	0.340	2.381 *	0.448	4.576 **	χ^2^ (203) = 427.198	χ^2^ (204) = 427.379 ^e^
Value from hedonic experience → Behavioral intentions	0.468	5.884 **	0.789	9.675 **	χ^2^ (203) = 427.198	χ^2^ (204) = 440.750 ^f^
Traveler satisfaction → Behavioral intentions	0.490	5.700 **	0.023	0.330	χ^2^ (203) = 427.198	χ^2^ (372) = 443.915 ^g^

Goodness-of-fit statistics for the baseline model for gender groups: χ^2^ = 427.198, *df* = 203, *p* < 0.001, χ^2^/*df* = 2.104, RMSEA = 0.062, CFI = 0.941, IFI = 0.942, TLI = 0.931. * *p* < 0.05, ** *p* < 0.01. Chi-square difference test: ^a^ Δχ^2^ (1) = 0.467, *p* > 0.05 (H8a: Not supported). ^b^ Δχ^2^ (1) = 3.760, *p* > 0.05 (H8b: Not supported). ^c^ Δχ^2^ (1) = 2.813, *p* > 0.05 (H8c: Not supported). ^d^ Δχ^2^ (1) = 3.502, *p* > 0.05 (H8d: Not supported). ^e^ Δχ^2^ (1) = 0.181, *p* > 0.05 (H8e: Not supported). ^f^ Δχ^2^ (1) = 13.552, *p* < 0.01 (H8f: Supported). ^g^ Δχ^2^ (1) = 16.717, *p* < 0.01 (H8g: Supported).

**Table 4:** The invariance model assessment for age groups.

Paths	High Age Group (*n* = 113)		Low Age Group (*n* = 177)		Baseline Model (Freely Estimated)	Nested Model (Equally Constrained)
	β	*t*-Values	β	*t*-Values		
Perceived inconvenience of tourism experience → Self-rated mental health	−0.416	−4.138 **	−0.235	−2.858 **	χ^2^ (203) = 411.828	χ^2^ (204) = 412.127 ^a^
Perceived inconvenience of tourism experience → Value from hedonic experience	−0.045	−0.595	0.005	0.066	χ^2^ (203) = 411.828	χ^2^ (372) = 412.042 ^b^
Self-rated mental health → Value from hedonic experience	0.774	9.707 **	0.592	8.191 **	χ^2^ (203) = 411.828	χ^2^ (372) = 422.819 ^c^
Self-rated mental health → Traveler satisfaction	−0.020	−0.113	−0.058	0.565	χ^2^ (203) = 411.828	χ^2^ (372) = 411.845 ^d^
Value from hedonic experience → Traveler satisfaction	0.105	0.593	0.463	4.355 **	χ^2^ (203) = 411.828	χ^2^ (372) = 414.999 ^e^
Value from hedonic experience → Behavioral intentions	0.570	6.978 **	0.632	8.394 **	χ^2^ (203) = 411.828	χ^2^ (372) = 412.489 ^f^
Traveler satisfaction → Behavioral intentions	0.434	4.936 **	0.205	2.937 **	χ^2^ (203) = 411.828	χ^2^ (372) = 416.663 ^g^

Goodness-of-fit statistics for the baseline model for age groups: χ^2^ = 411.828, *df* = 203, *p* < 0.001, χ^2^/*df* = 2.029, RMSEA = 0.060, CFI = 0.944, IFI = 0.945, TLI = 0.934. * *p* < 0.05, ** *p* < 0.01. Chi-square difference test: ^a^ Δχ^2^ (1) = 0.299, *p* > 0.05 (H9a: Not supported). ^b^ Δχ^2^ (1) = 0.214, *p* > 0.05 (H9b: Not supported). ^c^ Δχ^2^ (1) = 10.991, *p* < 0.01 (H9c: Supported). ^d^ Δχ^2^ (1) = 0.017, *p* > 0.05 (H9d: Not supported). ^e^ Δχ^2^ (1) = 3.171, *p* > 0.05 (H9e: Not supported). ^f^ Δχ^2^ (1) = 0.661, *p* > 0.05 (H9f: Not supported). ^g^ Δχ^2^ (1) = 4.835, *p* < 0.05 (H9g: Supported).

## Data Availability

The dataset used in this research are available upon request from the corresponding author. The data are not publicly available due to restrictions i.e., privacy or ethical.

## Appendix A

*Inconvenience of tourism experience* My overall experiences with halal foods offered in tourist places are uncomfortable. My overall experiences with halal facilities offered in tourist places are uncomfortable. My overall experiences with halal services and information in tourist places are uncomfortable. My overall experiences with staff/tourists/locals’ attire in tourist places are unpleasant and inconvenient. My overall experiences with staff/tourists/locals’ attitude toward Muslim travelers in many tourist places are unpleasant and disappointing.
*Self-rated mental health* During my travel to Korea, I feel that I can forget daily issues and anxiety. During my travel to Korea, I feel relaxed and calm. During my travel to Korea, I feel physically comfortable.
*Value from hedonic experience* My overall experiences while traveling to Korea are pleasant. My overall experiences while traveling to Korea are enjoyable. My overall experiences while traveling to Korea are interesting.
*Traveler satisfaction* Overall, I am satisfied with my travel experience to Korea as a halal-friendly destination. Overall, I have enjoyed myself while traveling to Korea as a halal-friendly destination.
*Behavioral intentions* I will say positive things about Korea as a tourist destination to other people. I am willing to revisit Korea in the future. I will make an effort to travel to Korea again in the future.
